# Arenavirus Budding: A Common Pathway with Mechanistic Differences

**DOI:** 10.3390/v5020528

**Published:** 2013-01-31

**Authors:** Svenja Wolff, Hideki Ebihara, Allison Groseth

**Affiliations:** 1 Institut für Virologie, Philipps Universität Marburg, Hans-Meerwein-strasse 2, 35043 Marburg, Germany; E-Mail: wolffs@staff.uni-marburg.de; 2 Laboratory of Virology, Division of Intramural Research, National Institute of Allergy and Infectious Diseases, National Institutes of Health, 903 South 4^th^ Street, Hamilton, MT 59840, USA; E-Mails: ebiharah@niaid.nih.gov (H.E.); allison.groseth@nih.gov (A.G.)

**Keywords:** arenavirus, budding, ESCRT pathway, matrix protein, nucleoprotein, late domain

## Abstract

The *Arenaviridae* is a diverse and growing family of viruses that includes several agents responsible for important human diseases. Despite the importance of this family for public health, particularly in Africa and South America, much of its biology remains poorly understood. However, in recent years significant progress has been made in this regard, particularly relating to the formation and release of new enveloped virions, which is an essential step in the viral lifecycle. While this process is mediated chiefly by the viral matrix protein Z, recent evidence suggests that for some viruses the nucleoprotein (NP) is also required to enhance the budding process. Here we highlight and compare the distinct budding mechanisms of different arenaviruses, concentrating on the role of the matrix protein Z, its known late domain sequences, and the involvement of cellular endosomal sorting complex required for transport (ESCRT) pathway components. Finally we address the recently described roles for the nucleoprotein NP in budding and ribonucleoprotein complex (RNP) incorporation, as well as discussing possible mechanisms related to its involvement.

## 1. Introduction

The *Arenaviridae* is a diverse and growing family of viruses, presently containing 24 recognized species [1] and several other proposed species [2,3,4,5,6,7,8,9,10,11,12,13,14], including many of considerable significance to human health (Table 1). Based on their antigenicity and phylogenetic analysis, and underscored by their geographical distribution, arenaviruses can be taxonomically divided into the Old World arenavirus (OWAV) and the New World arenavirus (NWAV) serocomplexes [2].

Among the OWAVs, both Lassa virus (LASV) and the recently identified Lujo virus (LUJV) are capable of causing hemorrhagic fever (HF) [7,15]. While the number of Lujo cases thus far reported has been minimal, LASV represents a major public health problem in Western Africa, where it has been estimated to cause 300,000–500,000 cases annually, resulting in 3,000–5,000 deaths [16]. In addition, while lymphocytic choriomeningitis virus (LCMV) usually causes asymptomatic illness in healthy individuals, in some cases it is known to cause aseptic meningitis, and while fatalities are rare, vertical transmission during pregnancy can have severe deleterious effects on the fetus. Further, severe infection has been reported in a few cases involving transplant patients [17], indicating that in immunocompromised individuals LCMV infection may pose additional risks. The remaining members of this serocomplex do not appear to be significant human pathogens. 

In the case of the NWAV serocomplex, viruses can be further divided into three clades (A, B and C) based on phylogenetic relationships [18], although recombination events between these clades are also possible. This is the case with Whitewater Arroyo virus (WWAV) as well as other North American arenaviruses, which are clade A/B recombinants [19]. Interestingly, this separation into distinct clades also corresponds to differences in receptor usage and disease phenotype [2]. The NWAVs are responsible for at least five distinct HFs [8,18], collectively referred to as South American Hemorrhagic Fevers (SAHFs), which are caused by Junìn (JUNV), Machupo (MACV), Chapare (CHPV), Guanarito (GTOV) and Sabiá (SABV) viruses. All of these viruses are members of the Clade B NWAVs and have been shown to use the Transferrin alpha receptor (TfR1) for entry into target cells [20,21]. While non-pathogenic Clade B members use the TfR1 orthologs of various other mammalian species [22], some evidence also exists that they can infect cells efficiently in a completely TfR1-independent manner [23]. NWAVs of Clade C have been shown to use α-dystroglycan as a receptor [24], similar to OWAVs [25].

In total arenaviruses cause at least seven distinct hemorrhagic fevers, making them the largest family of HF-causing viruses currently known. In addition, both the NWAVs and OWAVs contain a number of human apathogenic strains, which are in some cases quite closely related to the agents of HF disease. The basis for these marked differences in virulence among genetically closely related viruses remains unknown. Similarly, despite their significance for public health, many details of the arenavirus lifecycle, including the pathways used for virus morphogenesis and budding, remain poorly understood. However, in recent years significant progress has been made in this area and has revealed interesting commonalities in the mechanisms used by the various arenaviruses but also striking differences that remain to be fully explained from a mechanistic stand-point. Here we attempt to summarize the current state of our knowledge, including recent findings, in the field of arenavirus morphogenesis and budding.

**Table 1:** Viruses of the family *Arenaviridae*, their geographic distribution, reservoirs andassociated human diseases.

	Virus	Distribution	Reservoir	Human Disease
**Old World Arenviruses**	Dandenong virus*	Yugoslavia (?) Australia (?)	Unknown	Febrile illness with encephalopathy (transplant-related)
	Gbagroube virus*	Côte d'Ivoire	Mus (Nannomys) setulosus	None known
	**Ippy virus**	Central African Republic	*Arvicanthus spp.*	None known
	**Lassa virus**	Western Africa	*Mastomys natalensis*	Febrile illness, hemorrhagic fever in severe cases
	**Lymphocytic Choriomeningitis virus**	Worldwide	Mus musculus	Febrile illness, aseptic meningitis in severe cases
	**Lujo virus**	Zambia	Unknown	Hemorrhagic fever
	Luna virus*	Zambia	*Mastomys natalensis*	None known
	**Kodoko virus** *	Guinea	Mus (Nannomys) minutoides	None known
	Menekre virus*	Côte d'Ivoire	Hylomyscus *spp.*	None known
	**Merino Walk virus** *	South Africa	*Myotomis unisulcatus*	None known
	**Mobala virus**	Central African Republic	*Praomys jacksoni*	None known
	**Mopeia virus**	Mozambique	*Mastomys natalensis*	None known
	**Morogoro virus** *	Tanzania	*Mastomys spp.*	None known
**New World Arenaviruses**	**Allpahuayo virus**	Peru	*Oecomys spp.*	None known
	**Amapari virus**	Brazil	*Oryzomys gaeldi Neacomys guianae*	None known
	**Bear Canyon virus**	USA	Peromyscus californicus	None known
	Big Brushy Tank virus*	USA	*Neotoma albigula*	None known
	**Catarina virus** *	USA	*Neotoma micropus*	None known
	**Chapare virus**	Bolivia	Unknown	Hemorrhagic fever
	**Cupixi virus**	Brazil	*Oryzomys spp.*	None known
	**Flexal virus**	Brazil	*Oryzomys spp.*	Febrile illness(Lab-acquired)
	**Guanarito virus**	Venezuela	*Zygodontomys brevicauda*	Hemorrhagic fever
	**Junín virus**	Argentina	*Calomys musculinus*	Hemorrhagic fever
	**Latino virus**	Bolivia	*Calomys callosus*	None known
	**Machupo virus**	Bolivia	*Calomys callosus*	Hemorrhagic fever
	**Oliveros virus**	Argentina	*Bolomys spp.*	None known
	**Paraná virus**	Paraguay	*Oryzomys buccinatus*	None known
	**Pichinde virus**	Columbia	*Oryzomys albigularis*	None known
	**Pinhal virus**	Brazil	*Calomys tener*	None known
	**Pirital virus**	Venezuela	*Sigmodon alstoni*	None known
	**Real de Catorce virus** *	Mexico	*Neotoma leucodon*	None known
	**Sabiá virus**	Brazil	Unknown	Hemorrhagic fever
	**Skinner Tank virus** *	USA	*Neotoma mexicana*	None known
	**Tacaribe virus**	Trinidad	*Artibeus spp.* (bat)	Possible febrile illness (Lab-acquired)
	**Tamiami virus**	USA	*Sigmodon hispidus*	None known
	**Tonto Creek virus**	USA	*Neotoma albigula*	None known
	**Whitewater Arroyo virus**	USA	*Neotoma albigula*	Possible hemorrhagic fever

* proposed species not yet classified by the ICTV

## 2. Arenavirus Biology

**Figure 1:**
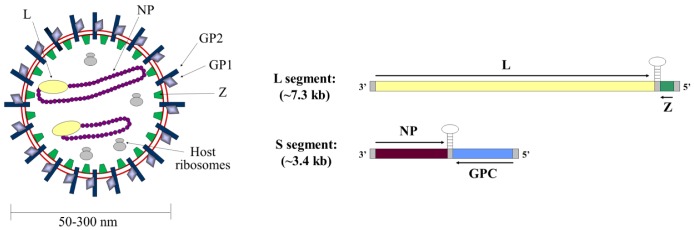
**Arenavirus genome and virion structure.** The general structure of an arenavirus particle (left panel) and the arenavirus genome (right panel) are shown. The mature viral glycoproteins GP1 and GP2 (shown in light and dark blue, respectively) are embedded in the viral envelope (shown in red). Beneath the viral envelope is the matrix protein Z (shown in green). The ribonucleoprotein complexes are composed of viral RNA (not visible) encapsidated by the nucleoprotein NP (shown in purple) and associated with the RNA-dependent RNA polymerase L (shown in yellow). In addition, arenavirus particles include host ribosomes (shown in grey) whose function remains unknown. The genes encoding for these viral proteins are arranged in ambisense orientation on the two genome segments. The small (S) segment encodes NP and the glycoprotein precursor (GPC), while the L segment encodes the polymerase L and the matrix protein Z.

Arenavirus particles are enveloped and highly pleomorphic, having a diameter of 50-300 nm (Figure 1). They contain a bi-segmented negative strand RNA genome that encodes for four viral proteins. The small genome segment (S segment, ~3.5 kb) contains genes for the surface glycoproteins (GP1 and GP2) and nucleoprotein (NP), while the large genome segment (L segment, ~7.5 kb) encodes the RNA-dependent RNA polymerase (L) and a multifunctional protein known as the small RING-finger protein (Z).

Together with NP and L the two ambisense genome segments are assembled into ribonucleoprotein complexes (RNPs), which serve as the templates for transcription and replication by the RNA-dependent RNA polymerase, L. Indeed, studies have shown that together NP and L represent the minimal protein requirements for transcription and replication of both NWAVs and OWAVs [26,27,28,29,30]. In addition to its role in viral transcription and replication, NP also functions as an interferon (IFN) antagonist [31]. NP interferes with interferon regulatory factor 3 (IRF3) activation, thereby inhibiting the induction of IRF3-dependent promoters, a process that is likely to be fundamental in the ability of arenaviruses to overcome the host innate immune response [32]. The glycoprotein GP is the only arenaviral surface protein and is synthesized as a precursor, GPC. It is first cleaved by signal peptidase to yield a stable signal peptide (SSP), which remains non-covalently bound to the glycoprotein [33,34,35]. Association with SSP is necessary for further cleavage of the glycoprotein into the receptor-binding ectodomain subunit, GP1, and transmembrane-spanning fusion competent subunit, GP2, by the proprotein convertase site 1 protease/subtilisin kexin isozyme-1 (S1P/SKI-1) during its transport through the secretory pathway [36,37,38]. This mature GP complex, consisting of GP1, GP2 and the SSP is located on the surface of virus particles [39].

**Figure 2:**
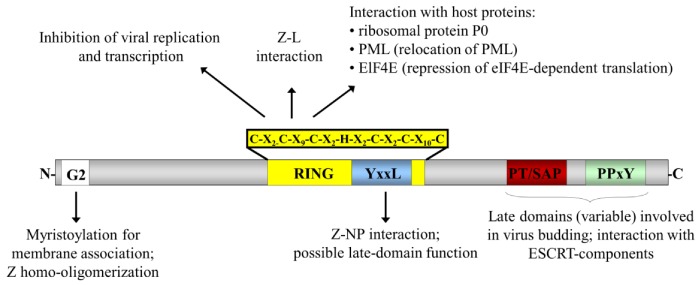
**Schematic representation of the arenavirus Z protein and the domains important for its interactions and functions.** The locations of the various functional domains in Z are indicated. The site of a myristoylated glycine residue at amino acid position 2 (G2) is indicated as a white box and the RING domain is indicated in yellow with the sequence of this domain indicated above. The various late domain motifs are indicated in colour: PT/SAP (red) and PPxY (green), as well as a putative late domain YxxL (blue). Known interaction partners and the functional roles for these domains are also indicated.

Despite being the smallest of the arenavirus proteins, with a size of only 89 to 103 amino acids, Z has been demonstrated to take part in a number of processes and interactions central to the viral lifecycle (Figure 2). Consistent with its numerous and varied biological roles, Z has been shown to participate in multiple interactions including directly interacting with the polymerase as well as with NP and GP, in addition to forming homo-oligomers [40,41,42]. The Z protein was first identified as a zinc-binding RING protein and found to be a negative regulator of genome transcription and replication [28,43]. However, since then it has also been shown to interact with several cellular factors, including the ribosomal P0 protein and the promyelocytic leukaemia protein (PML), which is redistributed to the cytoplasm after binding to Z [44,45,46]. Further, an interaction of Z with the eukaryotic translation initiation factor 4E (eIF4E), which is accompanied by repression of eIF4E-dependent translation in the host cell, was identified [47]. Additional studies have recently revealed an IFN regulating function for the Z protein of NWAVs (i.e. GTOV, JUNV, MACV and SABV), but not OWAVs (i.e. LASV and LCMV). This occurs as a result of its ability to bind to retinoic acid-inducible gene 1 (RIG-I) and thereby inhibit Type I IFN induction via the RIG-I signalling pathway [48], helping to overcome the innate immune response of the host cell. In addition to these regulatory functions, the Z protein of arenaviruses has been shown for several species to function as a matrix protein [49,50,51,52,53]. Characteristic for such proteins is their ability to mediate their own release in the form of virus-like particles, which resemble authentic virus particles in their morphology but lack the viral genomic material [54].

## 3. Virus Budding and Host Cell Sorting

The host cell membrane represents a significant barrier to the egress of newly formed or forming viral particles. In order to overcome this physical barrier many viruses have evolved to take advantage of host cell sorting pathways, in particular those directing vesicle formation into multi-vesicular bodies (MVBs). Thus for many enveloped viruses, including arenaviruses, it has been shown that they recruit the endosomal sorting complex required for transport (ESCRT) machinery of the host cell to facilitate their release [55,56]. However, there are also viruses whose release appears to be completely independent of the cellular ESCRT complexes [55,56]. The ability of viruses to subvert the cellular ESCRT machinery to drive viral budding likely derives from the fact that both of these processes involve budding being directed away from the cytoplasm, either into MVBs, in the case of vesicular budding, or into the extracellular space, in the case of virus budding.

In the uninfected host cell the ESCRT-system is involved in MVB vesicle formation and cytokinesis. It consists of six complexes (ESCRT-0, -I, -II, -III, Alix/AIP1 and Vps4) that are recruited sequentially to the sites of membrane remodelling and fission. While ESCRT-0 binds to and accumulates ubiquitinated cargo for delivery into the MVBs, the ESCRT-I and ESCRT-II complexes are recruited and co-assemble on the membrane to drive bud formation. ESCRT-III, which is responsible for final membrane scission, is recruited by binding to ESCRT-I and/or Alix/AIP1, which connects these complexes. Finally the AAA-type ATPase Vps4 mediates disassembly of the complex and its subsequent recycling [57]. During virus infection some of these ESCRT-complexes are targeted by the viral matrix protein, and in some cases also other viral proteins, to the sites of viral budding where they mediate the budding process of membrane enveloped particles using an analogous mechanism.

For many enveloped viruses it has been shown that the self-budding activity of their matrix protein is functionally dependent on the presence of late domain motifs within their sequences (reviewed in [56,57,58]). To date several late domain sequences have been reported (Table 2), with the tetrapeptide motifs PPxY, PT/SAP, and YxxL (x = any amino acid) appearing to be the most prevalent [55]. However, more recently θPxV (θ = hydrophobic amino acid) was identified as the functional late-domain sequence in simian virus 5 (SV5) [59] and although its interaction partner remains unknown, its identification serves to reinforce the possibility that other as yet unidentified late-domain motifs may also exist.

**Table 2:** Late-domain motif usage among different virus families.

Motif	Virus families using this motif (interacting proteins)	References
YxxL	*Arenaviridae* (NP, Z) *Retroviridae* (Gag) *Paramyxoviridae* (C, M)	[60,61,62,63,64]
PPxY	*Arenaviridae* (Z) *Filoviridae* (VP40) *Retroviridae* (Gag) *Rhabdoviridae* (M)	[52,65,66,67]
PT/SAP	*Arenaviridae* (Z) *Filoviridae* (NP, VP40) *Retroviridae* (Gag) *Rhabdoviridae* (M)*	[52,66,68,69,70]
θ PxV	*Paramyxoviridae* (M)	[59]

X indicates any amino acid, while θ indicates hydrophobic amino acids.

* not important for budding

**Figure 3:**
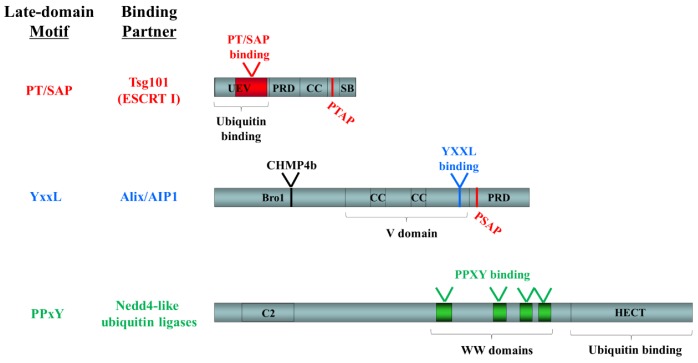
**Functional domains in known late-domain-interacting ESCRT-pathway components.** The following domains are indicated: UEV, ubiquitin E2 variant; PRD, proline-rich domain; CC, coiled-coil; SB, steadiness box; Bro1, BCK1-like resistance to osmotic shock; V, V domain; C2, conserved domain 2; WW, WW domain; HECT, homologous to the E6-AP carboxyl terminus. Late domain motifs and their binding sites are shown in color (PT/SAP, red; YxxL, blue; PPxY, green). Binding sites for other ESCRT pathway components are indicated in black.

Late-domain motifs promote viral budding by mediating the interaction of viral proteins with components of the cellular ESCRT-machinery or ESCRT-associated ubiquitin ligases, an observation that appears to hold true for a variety of virus families with which these studies have been conducted. The viral PT/SAP motif has been shown to recruit the ubiquitin-binding ESCRT-I-component tumor susceptibility gene 101 (Tsg101) to initiate viral budding [51,66,68,69,71]. This interaction occurs through an N-terminal ubiquitin E2 variant (UEV) domain, which is normally responsible for interaction of the ESCRT-I complex with ESCRT-0 and ubiquitinated cargo [72] (Figure 3). A C-terminally located PTAP motif in Tsg101 is also capable of binding to the UEV domain and in doing so blocks access of additional molecules, thus serving an auto-regulatory function [72]. For the PPxY motif, Nedd4-like ubiquitin ligases have been identified as an interaction partner [66,73,74] with interaction taking place between the PPxY tetrapeptide and a series of WW-domains in Nedd4-like proteins (Figure 3). Finally the MVB component Alix/AIP1 can also be recruited by viral matrix proteins and this occurs through binding of the YxxL motif to the V domain [61,64,75,76] (Figure 3). In addition Alix/A1P1 contains a C-terminal PSAP, which mediates its interaction with Tsg101, again through the UEV domain of Tsg101, and a Bro1 domain, which is responsible for interaction with CHMP4, an important ESCRT-III component [76].

## 4. Requirements for Arenavirus Budding

**Figure 4:**
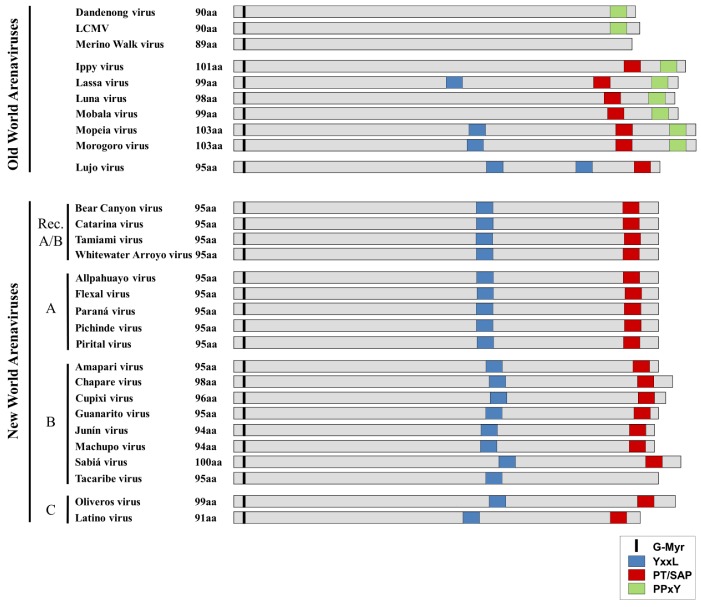
**Comparison of late-domain arrangements in Old World and New World Arenaviruses.** The locations of both putative and functional late domains for the various arenaviruses are shown. YxxL motifs are shown in blue, while PT/SAP motifs are shown in red and PPxY motifs are shown in green. The conserved glycine residue at which myristoylation occurs is indicated in black. Viruses are grouped according to their phylogenetic relationships (e.g. Old World/New World, Clade A, B, C or Recombinant (Rec. A/B)).

The Z protein has been shown for several arenaviruses to serve as the viral matrix protein providing the principle driving force for the budding of virus particles, and as such it is capable of forming virus like particles (VLPs) when expressed alone [54]. During budding, Z forms an inner layer beneath the viral envelope and is also able to interact with NP and GPC and to recruit them independently into viral particles. This NP-Z interaction is likely critical for incorporation of RNPs into progeny virions during the budding process. It has been directly shown for several arenaviruses that the budding activity of Z is linked to the presence of late domains and/or depends on the ESCRT pathway [49,51,53,71]. However, while one or more proline-rich late-domain (PT/SAP and/or PPxY) in the C-terminus and/or a YxxL motif located in the RING domain appear to be important for assembly and budding, the number and combination of the late domains within Z varies between different arenavirus species (Figure 4).

Sequence analysis of OWAVs shows that they generally contain either a PPxY motif alone or both a PPxY and a PT/SAP motif at their C-terminus. In addition, several of the OWAVs contain an additional YxxL motif in the RING domain. The exceptions to this are Merino Walk virus, which based on the available sequencing data does not appear to have any known late-domain motifs, and LUJV, which displays an unusual late domain motif arrangement more closely related to that of the NWAVs. This may reflect the fact that while LUJV is classified as belonging to the OWAVs, it is only distantly related to all other known OWAVs [7], which might explain its distinct late domain arrangement. In contrast to the OWAVs, nearly all NWAVs contain a single PT/SAP motif in the C-terminus, in addition to a YxxL in the RING domain. The only exception is TCRV, in which the canonical PT/SAP motif is replaced with an ASAP motif, which is not known to be functional in budding. 

Interestingly, despite the presence of multiple late domain motifs in some arenaviruses, the extent to which virus budding is dependent on each of these motifs seems to vary between viruses (Table 3). For OWAVs the proline-rich late-domains (PPxY and PT/SAP) have so far been shown to play the more significant role in Z-induced virus budding. However, while LCMV contains only a single PPxY motif in Z, which was identified as the major determinant responsible for budding [51], LASV Z contains both a PTAP and PPxY motif. In this case, while both motifs contribute to LASV particle release, PPxY has been reported to play the dominant role [52]. Interestingly, additional studies using siRNAs have revealed that LASV budding depends on the ESCRT-components Tsg101 and Vps4A/B but not Nedd4 or Alix/AIP1 [71]. This is despite the presence of a YxxL motif in the LASV Z protein, as well as the important role in LASV budding of the PPxY motif. Thus, this observation leaves the role, if any, of the YxxL late domain, as well as the identity of the ESCRT pathway interaction partner for the PPxY motif, unclear. For Mopeia virus (MOPV) it was also shown that the YxxL motif in Z does not contribute to its budding activity, but rather that it plays an important role in NP incorporation into Z-induced VLPs [60].

**Table 3:** Contributions of various matrix protein (Z) motifs and nucleoprotein(NP) to arenavirus budding.

		Influence on Budding					Ref.
		Z				NP	
		G2-myr	PPxY	PT/SAP	YxxL		
**Old World Arenaviruses**	**LCMV**	+++	+++	N/A	N/A	?	[51,77]
	**LASV**	+++	+++	+	?*	?	[52,78]
	**MOPV**	+++	?	?	-	-	[60,79]
**New World Arenaviruses**	**JUNV**	+++	N/A	+++	?	-	[49,80]
	**TCRV**	+++	N/A	N/A	-	+++	[53,80]

*LASV budding does not depend on Alix/AIP1

N/A, not applicable

**Figure 5:**
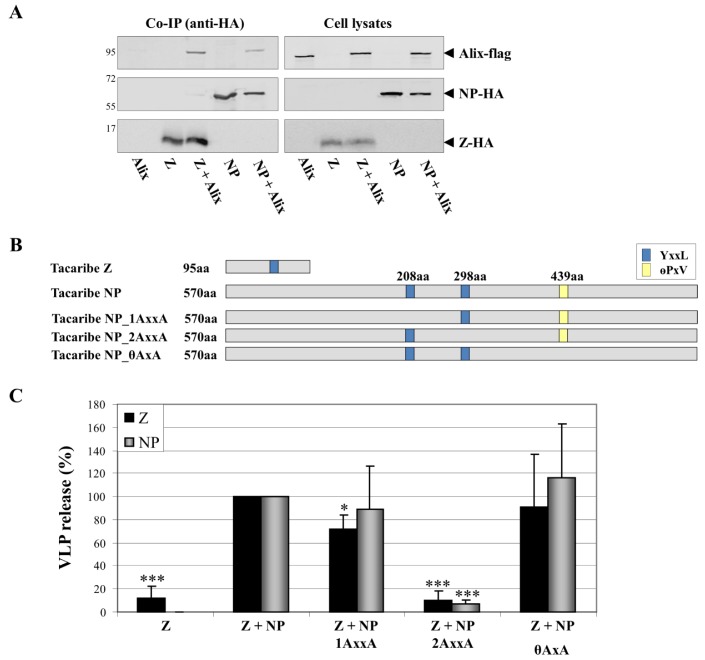
Role of NP late-domain motifs in the enhancement of TCRV Z-induced budding. **(A)** TCRV NP and Z both interact with the ESCRT-binding component Alix/AIP1. Co-immunoprecipitation studies using HA-agarose were performed 48 h after transfection of 293T cells with pCAGGS Alix-flag alone or in combination with pCAGGS TCRV NP-HA or TCRV Z-HA. **(B)** Schematic representation of TCRV NP and its putative late-domains. Mutants in which individual late domains are knocked-out (NP_1AxxA, NP_2AxxA, NP_θAxxA) by exchanges to alanine at the indicated positions are also shown. **(C)** TCRV Z-directed budding and the incorporation of NP late-domain mutants. VLPs were generated as previously described [80]. The release of Z in combination with NP was set at 100% and the relative budding efficiencies of Z alone or together with the various NP mutants shown in (B) were calculated. Release of Z in VLPs is shown in black while release of NP is shown in grey. Data represent the mean value and standard deviation of three independent experiments. The statistical significance was determined using Student’s t test. Asterisks indicate statistically significant differences (*p<0.05, **p<0.01, ***p<0.001).

For the NWAV JUNV, the release of chimeric VLP was shown to be heavily dependent on the PTAP motif [49], but the role of the YxxL motif in JUNV Z has so far not been analysed. Intriguingly, despite containing no canonical proline-rich late domains (the PSAP motif is replaced with an ASAP motif at the corresponding position), TCRV Z still functions as a matrix protein and initiates the release of VLPs in the absence of other viral proteins. However, both the ASAP and the YxxL motifs in TCRV Z appear to be expendable for the budding activity of Z [53,80], which is surprising given that TCRV Z can be shown by co-immunoprecipitation to bind to Alix/AIP1 (Figure 5A). Further, the release of TCRV VLPs is still dependant on the ESCRT pathway and has been shown to require Vps4A/B but not Tsg101 activity [53]. This finding also supports the lack of functional activity of the TCRV ASAP motif to mediate Z-Tsg101 interaction. Finally, recent studies have also revealed an essential role for NP in promoting TCRV budding, thereby providing further evidence that TCRV uses a budding mechanism distinct from that of other known arenaviruses, whose budding does not appear to be significantly influenced by NP [80].

## 5. Role of NP in Arenavirus Budding and RNP incorporation

NP has been described as an accessory factor for the budding of members of several virus families, including the paramyxoviruses [81,82], retroviruses [83] and filoviruses [69,84]. While the nucleocapsid (NC) domain of retroviruses interacts with Alix/AIP1 and is essential for Alix-mediated HIV release [85], Ebola virus VLP formation is driven by the major matrix protein VP40 but is significantly enhanced by the presence of NP [84]. For Marburg virus, NP was found to increase VP40-induced VLP release by recruiting Tsg101 via its PSAP motif [69], possibly in order to compensate for the absence of a PT/SAP motif in VP40 itself.

All evidence to date has clearly demonstrated that Z is the key determinant without which arenavirus budding cannot proceed. However, for TCRV Z, budding is considerably enhanced by the presence of NP [80], an effect not so far reported for other arenaviruses. This raises the possibility that, as for filoviruses, TCRV NP may be acting to recruit additional ESCRT complex components to the sites of budding. Indeed, sequence analysis shows that the NP of TCRV contains putative late domains of both the YxxL and the θPxV-type (Figure 5B), and that mutation of the YxxL motif at amino acid position 298 blocks enhancement of Z-mediated VLP release by NP (Figure 5C). Given that TCRV NP is able to functionally bind Alix-AIP1 (Fig 5A), this observation then suggests that NP may support virus budding by recruiting Alix/AIP1 to the sites of budding. This apparent difference in the mechanism of TCRV budding seems to correlate with its lack of any proline-rich late- domains in Z and the independence of budding on the Z protein YxxL late-domain motif [53,80]. This makes it interesting to speculate that other arenavirus Z proteins that lack known late domains, such as that of Merino Walk virus, might be similarly dependent on additional viral proteins to help promote budding.

Interaction of NP with Alix/AIP1, via its Bro-1 domain, has also been described for MOPV where, rather than enhancing budding, it was shown to mediate the incorporation of NP into Z-induced VLPs [60], suggesting that this interaction may be similarly responsible for the incorporation of RNPs into viral particles in the context of an infection. In MOPV the incorporation of NP into VLPs was shown to be facilitated by mutual interaction of Alix/AIP1 with both NP and Z, thus functioning as a “bridge” between these two viral proteins, which otherwise only weakly interact [60]. Since co-immunoprecipitation studies with TCRV also reveal an interaction of both NP and Z with Alix/AIP1 (Figure 5A) it is likely that an analogous interaction to that described for MOPV is also taking place between TCRV Z and NP. Indeed, such a mechanism would explain data indicating that mutational knock-out of the YxxL motif in TCRV Z blocks NP incorporation into VLPs [80]. However, it is interesting to note that while YxxL motifs in the RING domain are widely conserved among the NWAVs, MOPV is one of only a few OWAVs that possess a YxxL domain in Z (Figure 4). Thus it seems likely that alternative mechanisms to recruit RNPs, an essential step in virus infection, must also exist.

## 6. The Budding Pathway as a Potential Target for Therapeutics

Arenaviruses are serious public health concerns in many parts of the world and thus the development of vaccines and anti-viral therapies remains an important priority. An understanding of the exact mechanisms used for virus morphogenesis and release will help to offer new possibilities to combat viral infection by targeting these pathways. Such targets could include both the interactions among viral proteins (i.e. Z-NP interactions) and interactions between viral and host cell components. Emphasizing this potential, tetherin has recently been identified as a novel antiviral factor that is able to inhibit Z-mediated release of arenaviruses [86,87]. Tetherin or BST-2 (bone marrow stromal antigen 2) is an IFN-inducible membrane protein that was first shown to inhibit the release of HIV-1 [88,89]. For arenaviruses it was shown that the overexpression of tetherin reduced MOPV and LASV VLP production, as well as production of LASV virions [86]. Even if the exact mechanism by which tetherin antagonizes arenavirus budding remains unclear, the identification of this protein as an inhibitor of LASV Z-mediated budding [87] reinforces the importance of the budding pathway as a potential target for novel therapeutics.

In addition to the proline-rich and YxxL-type late-domains found within the matrix protein, N-terminal myristoylation also plays a decisive role in viral budding. This post-translational modification takes place at a glycine residue at position 2 (G2) in the Z protein sequence and is universally conserved among all arenaviruses (Figure 4). The myristoylation of Z is necessary for its binding to the plasma membrane, which is the site of arenavirus budding, and thus is also required for efficient viral release. For the OWAVs LASV, LCMV and MOPV, as well as for the NWAVs JUNV and TCRV it was shown that inhibition of myristoylation, either by mutation of the G2 residue or the use of an inhibitor, clearly represses Z-mediated budding [49,53,60,77,78], thus presenting another cellular pathway that could potentially be exploited by intervention strategies. 

Finally, the phosphatidylinositol 3-kinase (PI3K)/Akt pathway, which is involved in a variety of cellular processes [90], has also been shown to be important in the lifecycle of diverse viruses, including arenaviruses [91,92,93,94]. In the case of LCMV it was shown that inhibition of PI3K/Akt signalling, while not having an influence on viral entry, resulted in reduced viral RNA synthesis and the inhibition of viral budding [94]. As the PI3K/Akt pathway is involved in cellular vesicular trafficking, it has been suggested that the associated signalling might be needed for assembly of the ESCRT-complexes and, therefore, that an inhibition of this pathway might disrupt the budding process [94]. It has also been further speculated that the PI3K/Akt pathway might be involved in phosphorylation of LCMV and LASV Z [94]. However, despite these intriguing suggestions the exact mechanism by which the PI3K/Akt pathway contributes to arenavirus budding remains to be determined.

## 7. Conclusions

The formation of progeny virus particles and their release is an essential step in the lifecycle of a virus. In recent years significant progress has been made in the field of arenavirus morphogenesis and budding. The findings have revealed many commonalities in the budding mechanisms used by different arenaviruses, but also striking differences.

**Figure 6:**
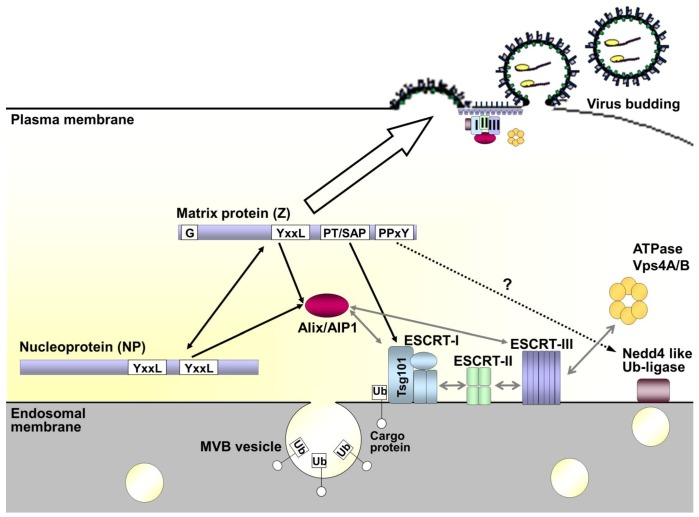
**Model of arenavirus budding and the role of the ESCRT pathway.** Under normal cellular conditions the ESCRT pathway is involved in vesicular trafficking of cargo proteins through multivesicular bodies (MVBs). This is facilitated by ubiquitination of the cargo protein, which mediates recognition by and sequential recruitment of the ESCRT-I, -II and -III complexes. Alix/AIP1 can also act to connect the ESCRT-I and -III complexes directly. Once recruited the ESCRT-III component is responsible for membrane scission, thereby releasing the newly formed vesicle. Finally, Vps4A/B mediates disassembly and recycling of the entire complex. During viral infection interaction of the matrix protein Z with components of the ESCRT pathway takes place through late domain motifs. The sequences are present in Z and vary between arenaviruses, but include some combination of PPxY, PT/SAP and/or YxxL sequences. These late domain motifs allow interaction with Nedd 4-like ubiquitin ligases, Tsg101 (a component of ESCRT-I) and Alix/AIP1, respectively. As a consequence of its interaction with these various ESCRT components and ESCRT-associated proteins, Z serves to redirect these components to the sites of budding at the cell membrane. This ability of Z to interact with the cell membrane is critically dependent on myristoylation of the glycine residue (G) at amino acid position 2 in the protein sequence. For some arenaviruses additional interaction of late-domain motifs in NP with Alix/AIP1 may further support budding, while also serving to recruit nucleocapsids into the forming virus particle through a direct Z-NP interaction.

The matrix protein Z of arenaviruses has been shown to provide the driving force for budding, an activity that depends on various distinct motifs that have been identified within the protein. Among these are an N-terminal myristoylation at glycine 2 (G2) of Z, which mediates binding of Z to the host cell plasma membrane, and various late domains (YxxL, PPxY and/or PT/SAP). These late domains are responsible for the recruitment of different components of the cellular ESCRT-machinery, normally involved in vesicle formation, to the budding-site where they initiate the release of membrane enveloped virions from the infected host cell (Figure 6). While the arrangement of late-domains in Z differs between the individual arenavirus species, interaction with components within the ESCRT-dependent budding pathway appears to be universal. While NWAVs mostly contain a single PT/SAP motif, OWAVs generally contain either a PPxY motif alone or both a PPxY and a PT/SAP motif at the C-terminus of the Z protein. The PT/SAP motif has been shown to mediate interaction with and recruitment of the ESCRT-I component Tsg101, however, an interaction partner for the PPxY motif of Z has yet to be identified. While interaction of the PPxY motif with a Nedd4-like ubiquitin ligase is likely, in the case of LASV, interaction with Nedd4 itself has been shown not to occur. TCRV Z appears to present a unique case in that it does not contain any proline-rich late domain at all. However, it still functions as a matrix protein. Perhaps due to this lack of any proline-rich late domains the ESCRT-dependent release of TCRV Z-induced particles is strongly enhanced by the presence of NP, raising the question of whether the recruitment of additional ESCRT-components might be assisted by late domains within NP (Figure 6). Such a mechanism appears, thus far, to be unique among the arenaviruses. Finally, recent studies of MOPV have revealed that the ESCRT-machinery might also facilitate recruitment of the viral nucleocapsid to the site of budding and its subsequent incorporation into particles through an interaction between Alix/AIP1 and both Z and NP. A similar mechanism also seems likely for TCRV; however, it remains unclear how OWAVs that lack a YxxL domain, and thus likely the ability to recruit Alix-AIP1, are able to incorporate their RNPs.

In summary, the current data reveal a budding mechanism for arenaviruses that is ESCRT-dependent, but involves a variety of different players depending on the virus species examined. Clearly there are also a number of unknowns and issues that need to be further investigated, especially given the significance of arenaviruses for public health, as a better understanding of the mechanisms and pathways used for virus morphogenesis might offer new possibilities for the development of antiviral therapies to combat arenavirus infection.
